# A deep neural network based regression model for triglyceride concentrations prediction using epigenome-wide DNA methylation profiles

**DOI:** 10.1186/s12919-018-0121-1

**Published:** 2018-09-17

**Authors:** Md. Mohaiminul Islam, Ye Tian, Yan Cheng, Yang Wang, Pingzhao Hu

**Affiliations:** 1 Department of Biochemistry and Medical Genetics, University of Manitoba, 745 Bannatyne Avenue, Winnipeg, MB R3E 0J9 Canada; 2 Department of Electrical and Computer Engineering, University of Manitoba, 66 Chancellors Cir, Winnipeg, MB R3T 2N2 Canada; 3 Department of Computer Science, University of Manitoba, 66 Chancellors Cir, Winnipeg, MB R3T 2N2 Canada; 4 George and Fay Yee Centre for Healthcare Innovation, University of Manitoba, 753 McDermot Avenue, Winnipeg, MB R3E 0T6 Canada; 5 Experimental Center, Northwest University for Nationalities, Chenggua, Lanzhou, 730030 Gansu China

## Abstract

**Background:**

Epigenetic modification has an effect on gene expression under the environmental alteration, but it does not change corresponding genome sequence. DNA methylation (DNAm) is one of the important epigenetic mechanisms. DNAm variations could be used as epigenetic markers to predict and account for the change of many human phenotypic traits, such as cancer, diabetes, and high blood pressure. In this study, we built deep neural network (DNN) regression models to account for interindividual variation in triglyceride concentrations measured at different visits of peripheral blood samples using epigenome-wide DNAm profiles.

**Results:**

We used epigenome-wide DNAm profiles of before and after medication interventions (called *pretreatment* and *posttreatment*, respectively) to predict triglyceride concentrations for peripheral blood draws at visit 2 (using pretreatment data) and at visit 4 (using both pretreatment and posttreatment data). Our experimental results showed that DNN models can predict triglyceride concentrations for blood draws at visit 4 using pretreatment and posttreatment DNAm data more accurately than for blood draws at visit 2 using pretreatment DNAm data. Furthermore, we got the best prediction results when we used pretreatment DNAm data to predict triglyceride concentrations for blood draws at visit 4, which suggests a long-term epigenetic effect on phenotypic traits. We compared the prediction performances of our proposed DNN models with that of support vector machine (SVM). This comparison showed that our DNN models achieved better prediction performance than did SVM.

**Conclusions:**

We demonstrated the superiority of our proposed DNN models over the SVM model for predicting triglyceride concentrations. This study also suggests that the DNN approach has advantages over other traditional machine-learning methods to model high-dimensional epigenome-wide DNAm data and other genomic data.

## Background

DNA methylation (DNAm) is a major epigenetic modification involving the addition of a methyl (CH3) group to the 5 position of cytosine residues in CpG (5′-cytosine-phosphate-guanine-3′) dinucleotide sequences by DNA methyltransferases to form 5-methylcytosine (5-mC). In humans, DNAm is very common and 5-mC is found in approximately1.5% of genomic DNA. The mutation of specific CpG sites is always associated with tissue-specific genes transcriptional repression, phenotype transmission and contributes to the development of different diseases by altering DNA accessibility and chromatin structure. The quantification of 5-mC content or global methylation in diseased or environmentally impacted cells could provide useful information for understanding of disease progression and mechanisms. DNAm variation has been proposed as an epigenetic biomarker for predicting the stage of disease, to determine a patient’s response to therapy, and to evaluate the prognosis [1].

Experimental and epidemiological evidences have reported that associate DNAm variations with blood lipid levels, such as high-density lipoprotein cholesterol, low-density lipoprotein cholesterol, triglycerides, and total cholesterol, by regulating the related gene of interindividual lipid levels. DNAm variations of CpG sites within *CPT1A* and *SREBF1* [2] gene promoters were linked with high triglycerides [2]. Triglyceride is a type of fat in human blood. Having a high concentration of triglycerides in human blood can increase our risk of heart, stroke, and other diseases. Many genetic loci have been identified by genome-wide association studies, but only a small proportion of interindividual variability of triglycerides has been explained by the genetic determinants. It is known that the level of triglycerides is heritable. Consequently, the development of new high-throughput genomic technologies makes it natural to extend these phenotypic prediction models to complex traits, such as triglyceride. Using DNAm profiles to predict disease phenotypic courses has not yet been explored in detail.

CpG sites with high interindividual variability of DNAm can indicate the possibility of different diseases, which means these CpG sites hold the patterns that are capable of discriminating between different phenotypes. As a heritable epigenetic mark, DNAm can explain the progress of many disease courses. Epigenome-wide DNAm has been used to predict different phenotypic traits. For example, Xu et al. [3] developed a novel support vector regression model for forensic age prediction by DNAm. Wilhelm [4] proposed a machine-learning model named Model-Selection–Supervised Principle Component Analysis (MS-SPCA) to predict different stages of cervical cancer using DNAm data. To avoid a potential overfitting problem in building these models, only a small handful of CpG sites are used in the models.

Newer machine-learning methods, such as deep neural network (DNN), can build a model using a large number of input features. These models show very promising results for several classification problems [5] in the field of computer vision. Unlike support vector machine (SVM), DNN does not require any handcrafted features and can automatically extract features from the raw input data. However, a SVM model will be likely overfitted when it is applied to methylation data with 450,000 CpG sites and only hundreds of samples because the underlying distribution is under sampled. In this paper, we propose DNN regression models for the prediction of triglyceride concentrations from multiple peripheral blood draws that are measured at different visits based on the individual’s epigenome-wide methylation profiles that are generated before and after medication interventions.

## Methods and materials

### Data sets

The data sets provided by GAW20 include epigenome-wide DNAm profiles and triglyceride concentrations (mg/dL) measured at baseline level (pretreatment) of visit 2 and changes in response to treatment with fenofibrate (posttreatment) at visit 4. The differential DNAm profiles were generated using the Illumina Infinium HumanMethylation450 BeadChip array. The beta value measuring the methylation level is expressed as a value between 0 and 1 in 993 participants of the Genetics of Lipid Lowering Drugs and Diet Network (GOLDN) study. It should be noted that there are only 499 participants with the posttreatment DNAm data. The GOLDN study recruited families with at least 2 siblings. For pretreatment data, we randomly selected 900 samples as the training set and another 93 samples as the test set; for posttreatment data, we randomly selected 400 samples as the training set and another 99 samples as the test set. We built the deep-learning models to predict triglyceride concentrations at visits 2 (pretreatment) and 4 (posttreatment) using the pretreatment DNAm data and at visit 4 using the posttreatment DNAm data. When we developed the model to predict posttreatment of triglyceride concentrations at visit 4 using pretreatment of DNAm data measured at visit 2, we only had 714 participants, from which 620 samples were randomly selected as the training set and the other 94 samples as the test set. The procedure to split the training and test sets was repeated three times. It should be noted that we did not use the “Answers” provided by GAW20 organizers during the analysis.

### Regression-based prediction models

#### Deep-learning regression model

**Fig. 1:**
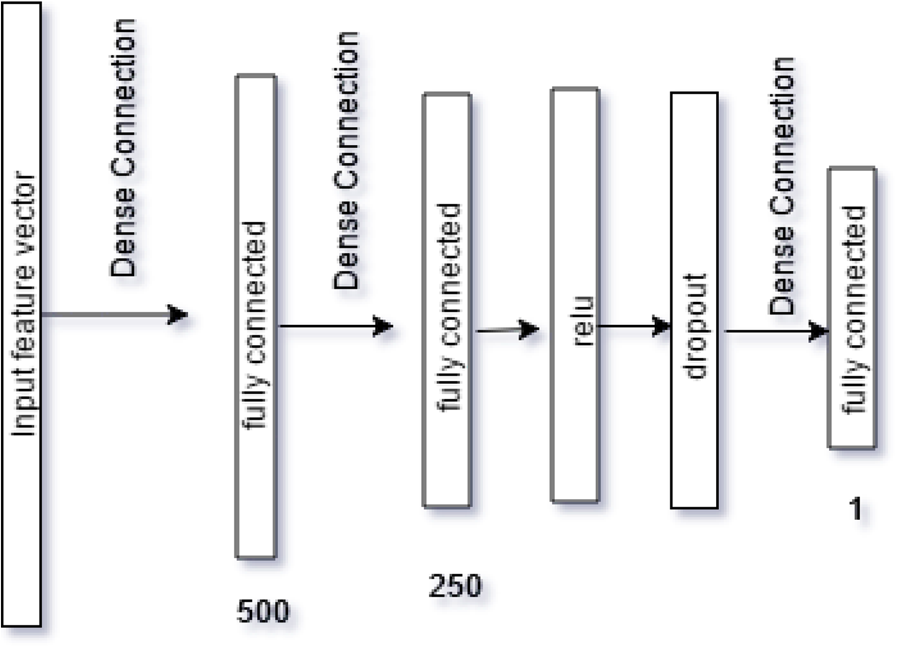
Proposed architecture of DNN. The numbers shown in the figure represent the size of the output of each layer

We proposed a DNN model (Fig. 1) to predict individuals’ triglyceride concentrations based on their epigenome-wide DNAm profiles provided by GAW20. DNN is an artificial neural network–based method, which is made up of a series of hidden layers between the input and output layers. DNN builds a hierarchy of features by producing high-level features from the low-level features. The bottommost layer (ie, input layer) of a DNN takes the raw input data and each next hidden layer learns an abstract form of the data from the previous layer.

The input of our proposed DNN network is a vector of the epigenome-wide DNAm profile of a given sample. Because the feature vector is quite high dimensional (> 450,000), we passed this input vector to two fully connected layers with different output sizes to reduce its dimension. These outputs can be thought as a matrix multiplication for getting high-level abstraction of the information in the input vector.

Because of the complex nonlinear relationship between triglyceride concentrations and genome-wide DNAm, we used a ReLU (rectified linear unit) layer followed by the second fully connected layer. The ReLU layer performs a ReLU thesholding function over the output of the second fully connected layer. The output of the ReLU layer is the nonlinear representation of the input to the network (see Fig. 1). The ReLU formularizes the relationship as:

1 $$ f(x)=\left\{\begin{array}{c}x,x\ge 0\\ {}0,x<0\end{array}\right. $$

Here,*x* represents the input to a neuron.

To provide generalization ability over the test data to the network we used a regularization technique called Dropout [6]. Dropout layers randomly drop out hidden neurons from the network. This technique allows the network to overcome the curse of overfitting because the network has to learn fewer parameters. Consequently, the output from the ReLU layer in our network was subjected to the dropout regularization technique by applying a dropout layer.

To get the final predictions of triglyceride concentrations we passed the output of the dropout layer to the last layer of the network, which is also a fully connected layer. We considered the score of this layer as the prediction of the network. Instead of using a greedy layer-wise (layer-by-layer) approach to train our network, we used a Euclidean loss layer to train our network in a backpropagation style. In this case, each layer of our DNN took an input and performed a transformation of the input to produce an output. This output was then used as an input to the next layer and so on until the loss layer was reached. This loss layer computed an error over its input data with respect to the ground truth value. Finally, a remedial gradient with respect to the error value was passed down to the DNN network to update its parameter values.

#### SVM model

SVM is a supervised learning algorithm that was initially developed to solve classification problems, but later was extended to solve regression problems [7]. SVM regression maintains all the key features that characterize the maximal margin theory and avoids difficulties of using linear functions in the high-dimensional feature space by transforming the optimization problem into dual convex quadratic programs. The loss function in SVM regression, which is used to penalize errors, usually leads to the sparse representation of the decision rule. This gives significant algorithmic and representational advantages over other regression methods.

#### Feature selection for DNN and SVM

For each sample, we have 463,995 CpG sites. As we know that CpG sites with high interindividual variability hold the most discriminative information [8], we defined the interindividual variability (*I*_*v*_) as the difference between 90th percentile and 10th percentile of the DNAm of a given CpG.

**Fig. 2:**
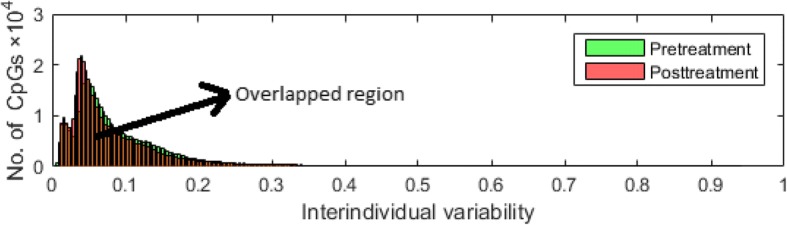
Distribution of inter-individual variability of DNAm for pretreatment and posttreatment

We built DNN models based on the selected CpG sites with *I*_*v*_ greater than or equal to different cutoffs of DNAm values (minimum [no filtering], first quartile, second quartile, mean, and third quartile). For each of these cutoff points we had 463,995, 348,223, 232,131, 165,817 and 116,057 CpG sites in the pretreatment data set and 463,995, 348,252, 231,901, 157,073 and 116,054 CpG sites in the posttreatment data set. The distributions of the interindividual variability *I*_*v*_ of DNAm in all CpG sites are shown in Fig. 2. Figure 2 clearly shows that the DNAm of a majority of the CpG sites has very small variation across samples. SVM will likely overfit the regression models if we use all 463,995 CpG sites to train the models. Consequently, we selected hundreds of the top CpG sites with larger interindividual variability of DNAm to build the SVM regression models.

#### Building the DNN

We used CAFFE, which is a C++ − based deep-learning library, to implement the DNN models (see Fig. 1) for different cutoffs of interindividual variability of DNAm (see Fig. 2). We trained all our DNN models using a learning rate (defined in the context of optimization, and minimizing the loss function of a neural network) of 0.000001, batch size (the number of training samples used in a single iteration/forward pass) of 10 and dropout ratio of 0.5.

#### Performance evaluation

We used root mean square error (RMSE) and Pearson correlation (Cor) methods to compare the performance of our DNN and standard SVM regression models. Gal and Ghahramani [6] also used RMSE to measure the performance of their deep-learning-based regression models. RMSEcan be calculated as follows:

2 $$ \mathrm{RMSE}=\sqrt{mean\left({\left(y-\widehat{y}\right)}^2\right)} $$

Here, *y* represents the observed triglyceride concentrations at different blood draws and $\widehat{y}$ represents the predicted triglyceride concentrations at different blood draws.

Cor was calculated between *y* and$\widehat{y}$. We performed three random splits between training and test data. The results of RMSE and Cor were averaged and their SDs were estimated. We used R package e1071 to build the SVM regression models (default parameters were used). Models with smaller RMSE or higher Cor are preferable and have better prediction performance.

## Results and discussion

**Fig. 3:**
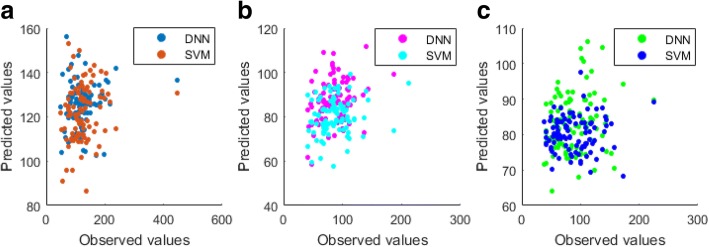
**a**: Pretreatment DNAm data to predict the triglyceride levels measured at visit 2; **b**: Pretreatment DNAm data to predict the triglyceride levels measured at visit 4; **c**: Posttreatment DNAm data to predict the triglyceride levels measured at visit 4

The *p* values of the Shapiro test of the log (base 2) of observed triglyceride concentrations in test sets from **Case A** (pretreatment DNAm data to predict the triglyceride levels measured at visit 2), **Case B **(pretreatment DNAm data to predict the triglyceride levels measured at visit 4), and **Case C** (posttreatment DNAm data to predict the triglyceride levels measured at visit 4), were 0.17, 0.25, and 0.25, respectively, suggesting that the observed triglyceride levels followed log-normal distribution. We performed the same procedure on their averaged predicted values from the three splits of training and test sets using the SVM models with largest Cor values (bold in Table 1) and the DNN model with largest Cor values (bold in Table 2) and the *p* values for **Case A**, **Case B**, and **Case C** were 0.09, 0.05, and 0.78, respectively, for SVM models, and 0.08, 0.14, and 0.59, respectively, for DNN models, which suggest that the predicted triglyceride levels using either DNN models or SVM models also follow log-normal distribution. The scatter plots of the observed and predicted triglyceride levels for **Case A**, **Case B**, and **Case C** are shown in Fig. 3.

**Table 1:** Performance of SVM models

Data^a^	EvaluationMetric^b^	Cutoffs^c^				
		100	200	300	400	500
1	RMSE	**90.3**(27.5)^d^	90.9(28.8)	90.9 (29.2)	90.8 (28.8)	95.8 (23.8)
	Cor	**0.13**(0.06)	0.11(0.12)	0.11 (0.14)	0.11 (0.14)	0.10 (0.13)
2	RMSE	**48.7**(13.7)	49.4(12.9)	49.0 (12.9)	**48.7** (12.8)	50.1 (14.3)
	Cor	**0.19**(0.08)	0.12(0.10)	0.15 (0.06)	0.17 (0.05)	0.04 (0.20)
3	RMSE	48.0(7.2)	47.6(7.0)	47.5 (6.9)	**46.9** (7.0)	47.0 (6.9)
	Cor	0.04(0.08)	0.07(0.09)	0.07 (0.10)	**0.13** (0.10)	0.12 (0.12)

^a^Data 1: Pretreatment DNAm data to predict the triglyceride levels measured at visit 2; Data 2: Pretreatment DNAm data to predict the triglyceride levels measured at visit 4; Data 3: Posttreatment DNAm data to predict the triglyceride levels measured at visit 4

^b^*RMSE* root mean square error, *Cor* Pearson correlation between observed and predicted values

^c^The top number of CpG sites selected based on interindividual variability

^d^The averaged RMSE or Cor value and their SD from the three splits of training and test sets. The bold value indicates the model has the best performance across a several number of selected CpG sites at the given DNAm data set and performance metric

**Table 2:** Performance of DNN models

Data	Evaluation Metric	Cutoffs^a^						
		Min	1st quartile	Mean	Median	3rd quartile	10kCpGs	1kCpGs
1	RMSE	**88.5** (26.3)	88.8 (25.6)	89.3 (25.7)	89.0 (27.3)	88.8 (26.1)	89.2 (25.9)	89.8 (26.4)
	Cor	0.19 (0.05)	**0.27** (0.08)	0.19 (0.09)	0.14 (0.11)	0.11 (0.10)	0.24 (0.02)	0.14 (0.11)
2	RMSE	48.5 (14.4)	48.4 (14.7)	**47.4** (13.7)	48.5 (14.3)	47.5 (13.8)	48.6 (12.9)	48.8 (13.0)
	Cor	0.23 (0.13)	0.10 (0.29)	**0.29** (0.07)	0.14 (0.19)	0.29 (0.07)	0.20 (0.11)	0.10 (0.14)
3	RMSE	48.5 (4.7)	48.7 (4.8)	48.5 (4.5)	**48.1** (3.5)	48.6 (4.6)	48.2 (5.0)	48.5 (5.3)
	Cor	0.17 (0.07)	0.18 (0.08)	**0.22** (0.13)	0.20 (0.12)	0.19 (0.08)	0.17 (0.06)	0.16 (0.04)

^a^The selected CpG sites with interindividual variability greater than or equal to different cutoffs of DNAm values (minimum [no filtering], first quartile, second quartile, mean, and third quartile) as well as the top 10,000 CpG sites (10kCpGs) and top 1000 CpG sites (1kCpGs)

The prediction results (RMSE and Cor) of the SVM and our DNN models using different number of CpG sites with larger interindividual variability of DNAm are shown in Tables 1 and 2, respectively. In general, the number of CpG sites used in each model (DNN or SVM) has little effect on the prediction performance as measured by RMSE of the triglyceride concentrations measured at a specific visit. However, the number of CpG sites used in each model (DNN or SVM) does impact the prediction performance measured by Cor. For example, the SVM models with a larger number of CpG sites (eg, 500) have poorer performance than those with a smaller number of CpG sites (eg, 100; see Table 1), but the DNN models with a larger number of CpG sites (eg, 165,817) have much better performance than those with a smaller number of CpG sites (eg,1000; see Table 2). Comparison of the performances of our DNN models (see Table 2) with those of SVM models (see Table 1) shows that our proposed models have a lower RMSE and a higher correlation between predicted and observed triglyceride concentrations, which suggests that our DNN models have better prediction performance than do the SVM models.

Overall, using DNN and SVM models to predict triglyceride concentrations with DNAm profiles has worse performance at visit 2 than at visit 4. Remarkably, our DNN results (the averaged RMSE and Cor) show that the performances of using pre- and posttreatment DNAm to predict triglyceride levels at visit 4 are similar. For example, the best performance of using pretreatment DNAm to predict triglyceride levels at visit 4 is 47.4 for RMSE and 0.29 for Cor while the best performance of using posttreatment DNAm to predict triglyceride levels at visit 4 is 48.1 for RMSE and 0.22 for Cor. Furthermore, this finding also shows that pretreatment DNAm has slightly better capability to predict triglyceride levels than posttreatment DNAm at visit 4. These results have two potential implications: (a) the variation of DNAm may not be altered greatly as a result of treatment, and (b) early DNAm variation could predict the internal response of the individuals to lipid-lowering drugs. Consequently, DNAm may have a long-term effect on genome sequence under exposure to early environmental experiences that were associated with stable changes in the gene expression that emerged in the initial stage of disease and were sustained into later stages. Much research [9] supports the long-term epigenetic effect on genomes, making the DNAm profile usable as the epigenetic marker to predict development and prognoses of diseases.

## Conclusions

This study proposed a DNN architecture for predicting triglyceride concentrations, a complex phenotypic trait, using epigenome-wide DNAm profiles measured at different patient visits for blood draw. The new model framework has advantages over some traditional learning algorithms (such as SVM), which are prone to overfitting when the input data are quite high dimensional. We showed that DNAm profiles measured at pretreatment and posttreatment have a better capability to predict triglyceride concentrations measured from blood drawn at visit 4 than do DNAm profiles measured at pretreatment to predict triglyceride concentrations measured from blood drawn at visit 2. We also found that DNAm profiles measured at pretreatment can predict triglyceride concentrations measured from blood drawn at visit 4 more accurately than DNAm profiles measured at posttreatment, which suggests a long-term epigenetic effect on phenotypic traits. The limitations in the study are that the proposed model neither considered the familial relationships of the participants in the study nor explored the usefulness of the available genetic data to predict the triglyceride levels. We will investigate whether the DNN model is sensitive to the familial structure and integrate both genetic and methylation data to predict triglyceride levels in the future.
